# A new species and two newly recorded species of the genus *Metopomyza* Enderlein from northern China (Diptera, Agromyzidae) with a worldwide key and checklist

**DOI:** 10.3897/zookeys.1290.199747

**Published:** 2026-08-18

**Authors:** Xin-Ting Fu, Li Shi

**Affiliations:** 1 College of Horticulture and Plant Protection, Inner Mongolia Agricultural University, Hohhot, Inner Mongolia, 010018, China College of Horticulture and Plant Protection, Inner Mongolia Agricultural University Hohhot China

**Keywords:** Morphological structure, new record, phylogenetic tree, Phytomyzinae, taxonomy

## Abstract

A new species, *Metopomyza hebeiensis***sp. nov**., together with two newly recorded species for China (i.e., *M. laeta* and *M. scutellata*), are documented and illustrated in this study; a worldwide identification key and checklist of species included in the genus are also provided. The Maximum-Likelihood and Neighbor-Joining phylogenetic trees based on 48 sequences confirm that our specimens belong to *M. scutellata*, but this does not prove that *M. scutellata* and *M. junci* are conspecific.

## Introduction

*Metopomyza* Enderlein, 1936 is a small genus of Agromyzidae (Diptera) with 23 species worldwide (see a checklist below) distributed in the Palaearctic, Nearctic, and Oriental regions (von Tschirnhaus and Groll 2024a, 2024b). *Metopomyza griffithsi* Sehgal (Canada, Alberta) was treated as *Didymyza* Sasakawa by Sasakawa (2020), but we follow von Tschirnhaus and Groll (2024a) in not recognizing this genus. *Metopomyza taipingensis* recorded firstly by Shiao and Wu (1996) from China (Taiwan island) was reassigned to Cerodontha (Icteromyza) by Zlobin (2007b) based on its male genitalia. The genus was also reported from the Afrotropical and Neotropical regions with undescribed species (Lonsdale and von Tschirnhaus 2021; Boucher et al. 2025). However, so far there has been no distribution record from the Australian/Oceanian region. The species *Metopomyza xanthaspis* (Loew, 1858) was reported from China (Xinjiang) in 2007 (https://portal.boldsystems.org/record/CNCLP207-09). This was the only existing record of the genus in China before the commencement of our research.

**Figure 1.**
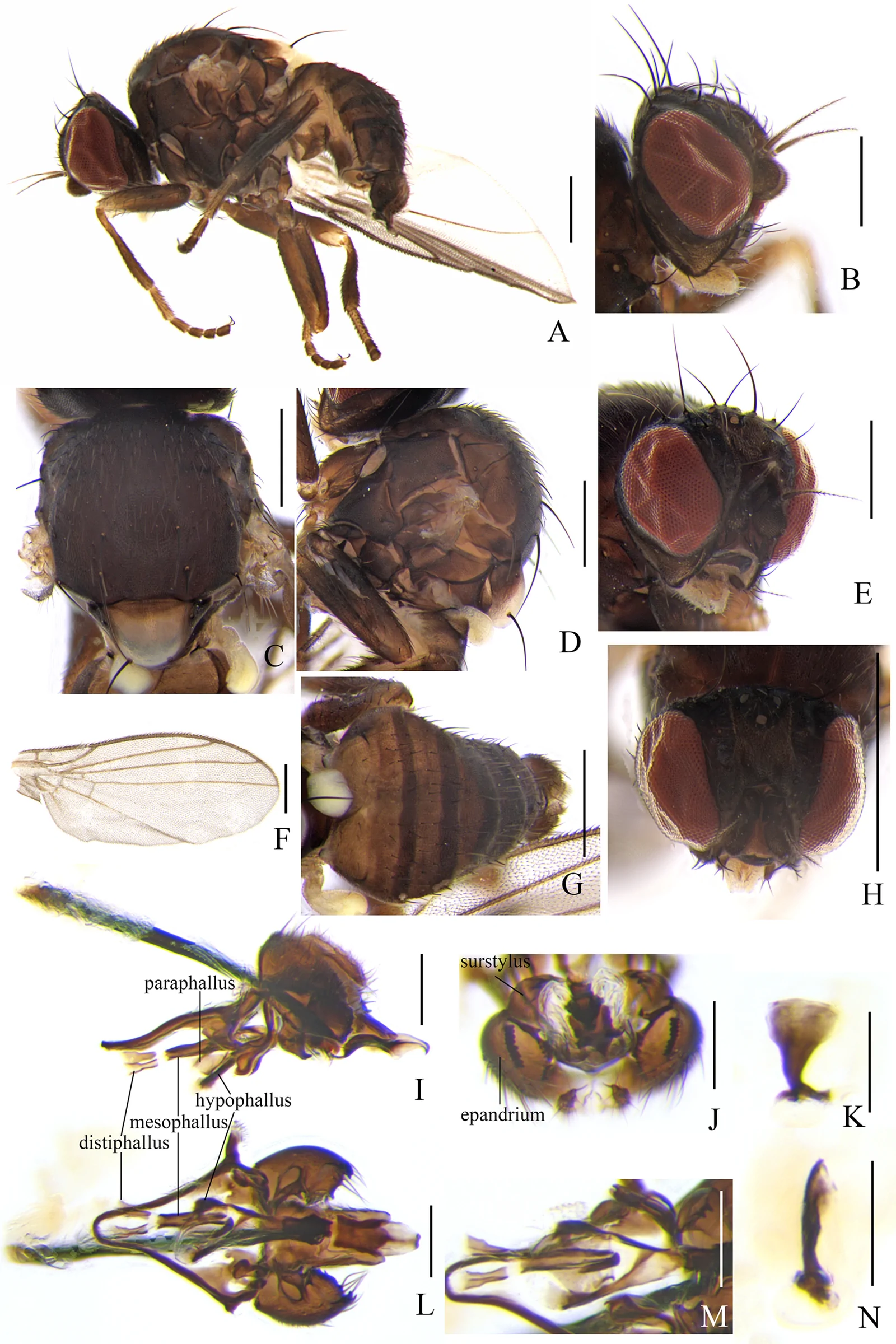
*Metopomyza hebeiensis* sp. nov. Holotype male. **A**. Habitus, lateral view; **B, E, H**. Head, lateral, anterolateral, and dorsal view; **C, D**. Thorax, dorsal and lateral view; **F**. Wing; **G**. Abdomen, dorsal view; **I, L**. Genitalia, lateral and ventral view; **J**. Epandrial complex, ventral view; **M**. Phallic complex, ventral view; **K, N**. Ejaculatory apodeme, lateral and ventral view. Scale bars: 0.25 mm (**A–H**); 0.1 mm (**I–N**).

The species of the genus *Metopomyza* can be distinguished by the following characters: adults largely black; fronto-orbital plate brownish black, sometimes largely yellow, relatively wide and pronounced; inner edges of fronto-orbital plates and anterolateral edges of ocellar triangle forming an M-shaped pattern with two sharp angles in dorsal view (Lonsdale and von Tschirnhaus 2021), which sometimes occurs in other genera such as *Cerodontha* Rondani; first flagellomere small, showing an angulate tendency (sometimes subquadrate); mesonotum black, with 2–4 strong postsutural *dc*, presutural *dc* very short or absent; *acr* in 2–7 irregular rows; notopleuron usually black, sometimes yellow; scutellum entirely or partly yellow, or entirely brown to black (such as *M. nigrina*); wing with costa extending to apex of M_1_ or ending between R_4+5_ and M_1_; legs largely black, knees often yellow (in *M. flavipes* femora yellow), sometimes tarsi completely yellow; epandrium with a row of blunt tubercle-like setae on ventral margin and one or two rows of blunt tubercle-like setae on inner surface (Figs 1J, 3J, 4I, 6M); surstylus flap-shaped, completely separated from epandrium by suture, inner margin usually concave, upper corner with a few setulae, lower corner with 1–3 tubercle-like setae (Figs 3L, 4L, 6N) (Zlobin 1995; Lonsdale and von Tschirnhaus 2021).

Herein a new species and two newly recorded species of the genus *Metopomyza* are reported from northern China. The photographs of habitus and terminalia of these species are presented with a key and checklist of the genus *Metopomyza* worldwide.

## Materials and methods

A survey of Diptera was conducted for the first time from July to August 2022–2023 in the northern primitive forest region of the Greater Khingan Mountains in Inner Mongolia, China. The 21 sampling points were selected with 42 Malaise traps, and a total 237,320 specimens of 69 families of insects were captured in this survey. Among them, 175 species of 106 genera of 30 families in Diptera were recorded till December in 2025, and others are still in progress. Another survey of insects was conducted for the first time from July to August 2023–2024 in ‌the semi-humid and semi-arid regions in northern China. The 14 sampling points were selected with 132 Malaise traps and a total of more than 282,900 specimens of 186 families of Diptera were captured in this survey. The identification and classification of Diptera are in progress. The above-mentioned Malaise traps were spaced more than 50 m apart, and the bottles were changed every 15 days. Insect samples were stored in 75% ethanol in the field and then transferred to anhydrous ethanol for long-term storage in a low-temperature freezer of the laboratory, with collection location, vegetation type and collection time recorded.

To prepare the genitalia, the apex of the abdomen was excised and subjected to maceration in warm lactic acid for 20–25 min. Following this treatment, specimens were rinsed with purified water to facilitate dissection and morphological analysis. After examination in glycerin, the genital structures were transferred and stored in a microvial containing glycerin for long-term preservation.

Specimens were examined with a Nikon 1270 dissection microscope. Images of adult individuals and genitalia were captured with a DMC6200 digital camera mounted on a Leica M205FA dissecting microscope. Sequential images were subsequently combined into composite montages using Leica Application Suite X 5.0.2.24429 software. Final image processing, including optimization and editing, was performed using Adobe Photoshop CS 6.0® to enhance clarity and consistency.

The type specimens of the new species and other studied specimens are deposited in the Insect Collection of Inner Mongolia Agricultural University, Hohhot, Inner Mongolia, China (**IMAU**). The two new records for China are indicated by the symbol # in the sections of distribution.

The general terminology follows Lonsdale (2021) and Fu et al. (2026). Abbreviations of morphological terms used in the text: ***acr***—acrostichal setulae, ***dc***—dorsocentral setae, ***ori***—inferior fronto-orbital seta, ***ors***—superior fronto-orbital seta, **M_1_**—first branch of vein Media, **M_4_**—fourth branch of vein Media, ***r-m***—crossvein radial-medial, ***dm-m***—crossvein discal medial.

For DNA extraction of adult specimens of *Metopomyza scutellata* (abdomen mostly brown and tergites 2–4 broadly yellow laterally), living specimens were soaked in absolute ethyl alcohol after being captured in the field and stored at −20 °C prior to DNA extraction. Specimens were identified based on their morphological features. The specimen’s DNA was extracted from the muscles of head and thorax individually by TIANamp Micro DNA Kit (Tiangen, Beijing, China) following the manufacturer’s instructions. The remaining tissue and DNA evidence were preserved under −20 °C in the Insect Collection of Inner Mongolia Agricultural University (Hohhot, China).

A fragment of 654 bp of the barcode region of the mitochondrial COI gene was generated by PCR amplification using the primer pair LCO1490 and HCO2198 (Gutiérrez-López et al. 2015; Kirchgatter et al. 2020) and following the PCR response condition with a total reaction volume of 25 μL containing 12.5 μL TIANGEN 2x Taq PCR Master Mix, 9.5 μL ddH_2_O, 1 μL 10 μM forward and reverse primers (synthesized by the Sangon Biotech (Shanghai) Co., Ltd) and 1 μL DNA template and a thermocycling procedure (Forward: an initial denaturation at 95 °C for 5 min, then 94 °C denaturation 30 s, at annealing 51 °C for 30 s, extension at 72 °C for 1 min, by 35 cycles, then with a final extension at 72 °C for 3 min, then stored at 4 °C; Reverse: an initial denaturation at 95 °C for 3 min, then 94 °C denaturation 30 s, at annealing 53 °C for 45 s, extension at 72 °C for 1 min, by 35 cycles, then with a final extension at 72 °C for 7 min, then stored at 4 °C).

For sequencing, alignment, and phylogenetic analysis, the amplified fragments were sequenced in both directions by the Sangon Biotech (Shanghai) Co., Ltd. The sequences from the company were assembled by SnapGene version 6.0.2. and were aligned with the sequences in GenBank, and then the COI sequence of *Metopomyza scutellata* was deposited in the GenBank database (accession number PZ742162).

To ‌explore the phylogenetic relationships between *Metopomyza scutellata* and *M. junci*, 44 sequences of nine species of *Metopomyza* were downloaded from the GenBank and BOLD databases. In addition, four species of *Liriomyza* and *Phytoliriomyza* were used as outgroups to root the tree. Alignments of the sequences were then inspected and edited in MEGA version 12.1.2.

Both the Maximum-Likelihood (ML) and Neighbor-Joining (NJ) were used to construct the phylogenetic tree based on above-mentioned 48 sequences. The ML and NJ tree were constructed using a standard bootstrap method with 1000 replicates in MEGA version 12.1.2 under GTR model with Gamma-Distributed rate variation across sites.

## Taxonomy

### Metopomyza hebeiensis sp. nov.

Taxon classificationAnimaliaDipteraAgromyzidae

EA84B242-956D-5AF3-9D98-918DB272700D

https://zoobank.org/ECD56A51-0373-4E89-B0F0-3B0D03422629

Figs 1A–N, 2A–L

#### Type material.

***Holotype***. • ♂ (IMAU; genitalia dissected), China, Hebei Province, Chengde City, Weichang County, Saihanba National Forest Park, Dahuanqi Town, *Pinus tabuliformis*, malaise trap 4, 42°22'13.88"N, 117°34'11.55"E, 1,466 m, 09.VII.2023, leg. Qi Liu, Sheng Jin. ***Paratypes***. • 3♀ (IMAU; 1♀ terminalia dissected), same data as for holotype.

#### Diagnosis.

Wing length 1.4–1.6 mm. Fronto-orbital plate matt black, slightly more than 1/2 width of frons at level of posterior *ors*. Gena deep, ~1/3 height of eye, forming broad ring below eye. Mesonotum with 0+2 *dc*; intra-alar setae absent. Wing with the ultimate and penultimate sections of M_4_ in proportion of 3.2:1. Abdomen entirely brown. Male genitalia: surstylus discrete and directed inward; two stout tubercle-like setae on posteroventral corner and one tubercle-like setae on anteroventral corner; hypophallus directed anteriorly; distiphallus conspicuously dilated on distal half, ~1/2 length of mesophallus.

#### Description.

**Figure 2.**
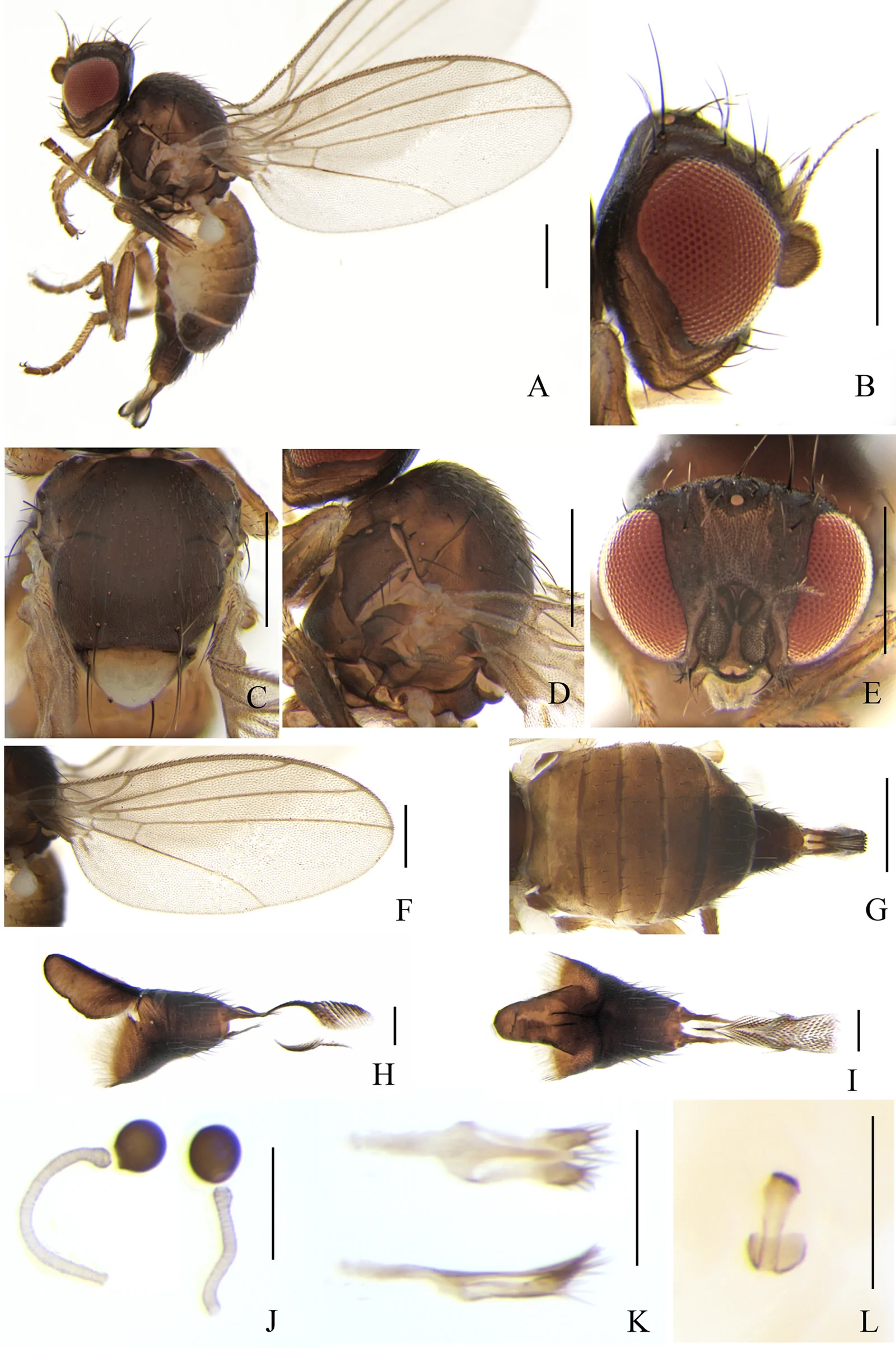
*Metopomyza hebeiensis* sp. nov. Paratype female. **A**. Habitus, lateral view; **B, E**. Head, lateral and anterior view; **C, D**. Thorax, dorsal and lateral view; **F**. Wing; **G**. Abdomen, dorsal view; **H, I**. Oviscape and ovipositor sheath, lateral and ventral view; **J**. Spermathecae; **K**. Proctiger, ventral and lateral view; **L**. Ventral receptacle, ventral view. Scale bars: 0.25 mm (**A–G**); 0.1 mm (**H–L**).

**Male** (Fig. 1A–N). Body length 1.5 mm; wing length 1.4 mm. Female (Fig. 2A–L). Body length 1.4–1.5 mm; wing length 1.4–1.6 mm.

***Head*** (Figs 1B, 1E, 1H, 2B, 2E) mostly dark brown. Frons brown, distinctly projecting above eye in lateral view; fronto-orbital plate matt black with inner margin yellow, slightly more than 1/2 width of frons at level of posterior *ors*; two *ori* inclinate and two *ors* reclinate; orbital setulae relatively long, conspicuously reclinate in a single row. Ocellar triangle black and well-sclerotized, ocellar setae and posterior *ors* equal in length. Lunule brownish black. First flagellomere subquadrate, more rounded below; arista microscopically pubescent. Gena brownish black, ~1/3 height of eye, forming conspicuously broad ring below eye. Clypeus black and broad; palpus dark brown.

***Thorax*** (Figs 1C, 1D, 2C, 2D) mostly black. Mesonotum dark brown, moderately shiny, 0+2 *dc*, *acr* in 6 irregular rows; one presutural and two strong postsutural supra-alar setae; intra-alar setae absent. Notopleuron brown. Scutellum largely yellow, black laterally to base of basal scutellar setae. Legs mostly brownish black with all knees yellow; tarsi brown. Wing (Figs 1F, 2F): costa extending M_1_ with 2^nd^ (between R_1_ and R_2+3_), 3^rd^ (between R_2+3_ and R_4+5_) and 4^th^ (between R_4+5_ and M_1_) sections in proportion of 3.2:1.5:1; ultimate and penultimate sections of M_1_ in proportion of 5:1; *r-m* slightly beyond middle of discal cell; ultimate and penultimate sections of M_4_ in proportion of 3.2:1. Calypter yellow with fringe and margin brown.

***Abdomen*** (Figs 1G, 2G) entirely brown. Male genitalia (Fig. 1I–N): epandrium with a row of 8–9 short tubercle-like setae on ventral corner, and two rows of 5–6 short tubercle-like setae on inner surface (Fig. 1J); surstylus discrete and directed inward, with two stout tubercle-like setae on ventral corner and one tubercle-like seta on anteroventral corner (Fig. 1J); hypophallus directed anteriorly (Fig. 1I); mesophallus relatively long, widest basally in lateral view; distiphallus conspicuously dilated on distal half, ~1/2 length of mesophallus; ejaculatory apodeme (Fig. 1K, N) slightly narrow with clear blade margin and unpigmented base of sperm pump.

**Female**. Wing with *r-m* not beyond middle of discal cell (Fig. 2F); abdominal tergites 2–4 yellowish laterally (Fig. 2G); other external characters (Fig. 2A–E) same as for male except for the female terminalia. Terminalia (Fig. 2H–L): spermathecae (Fig. 2J) dark brown, nearly circular; spermathecal duct weakly sclerotized, slightly dilated near spermathecae; ventral receptacle symmetrical; proctiger (Fig. 2K) 4× as long as maximum width.

#### Chinese name.

He bei e qian ying (河北额潜蝇).

#### Distribution.

China (Hebei).

#### Etymology.

The new species is named after the type locality Hebei province in China.

#### Remarks.

The new species is similar to *M. scutellata* in the following characters: head and thorax mostly dark brown, frons brown, fronto-orbital plate with two *ori* and two *ors*. But it can be distinguished by the following characters: mesonotum with two postsutural *dc*; abdominal tergites dark brown, without yellow posterior margin; hypophallus directed anteriorly (Fig. 1I). In *M. scutellata*, mesonotum with three postsutural *dc*; abdominal tergites with yellow lateral and posterior margins; hypophallus directed ventrally.

### Metopomyza laeta

Taxon classificationAnimaliaDipteraAgromyzidae

Sasakawa, 1955

4E5F5ED8-09C1-5F3D-BBFA-E52BD1C8AFDA

Fig. 3A–N

Metopomyza laeta Sasakawa, 1955: 116; Sasakawa 1961: 411–412; von Tschirnhaus 1981: 332; Zlobin 1995: 159; Černý 2018: 129.

#### Specimens examined.

• 1♂ (IMAU; genitalia dissected), China, Inner Mongolia, Genhe City, Mangui Town, the northern primitive forest region of Greater Khingan Mountains, Wulonggan forestry center near impounding reservoir, unburned area, Pinus sylvestris var. mongholica malaise trap 19 (two meters above the ground), 52°47'47.98"N, 120°55'03.45"E, 816 m, 16.VIII.2023, leg. Qin-Jianrong Liu.

#### Diagnosis.

**Figure 3.**
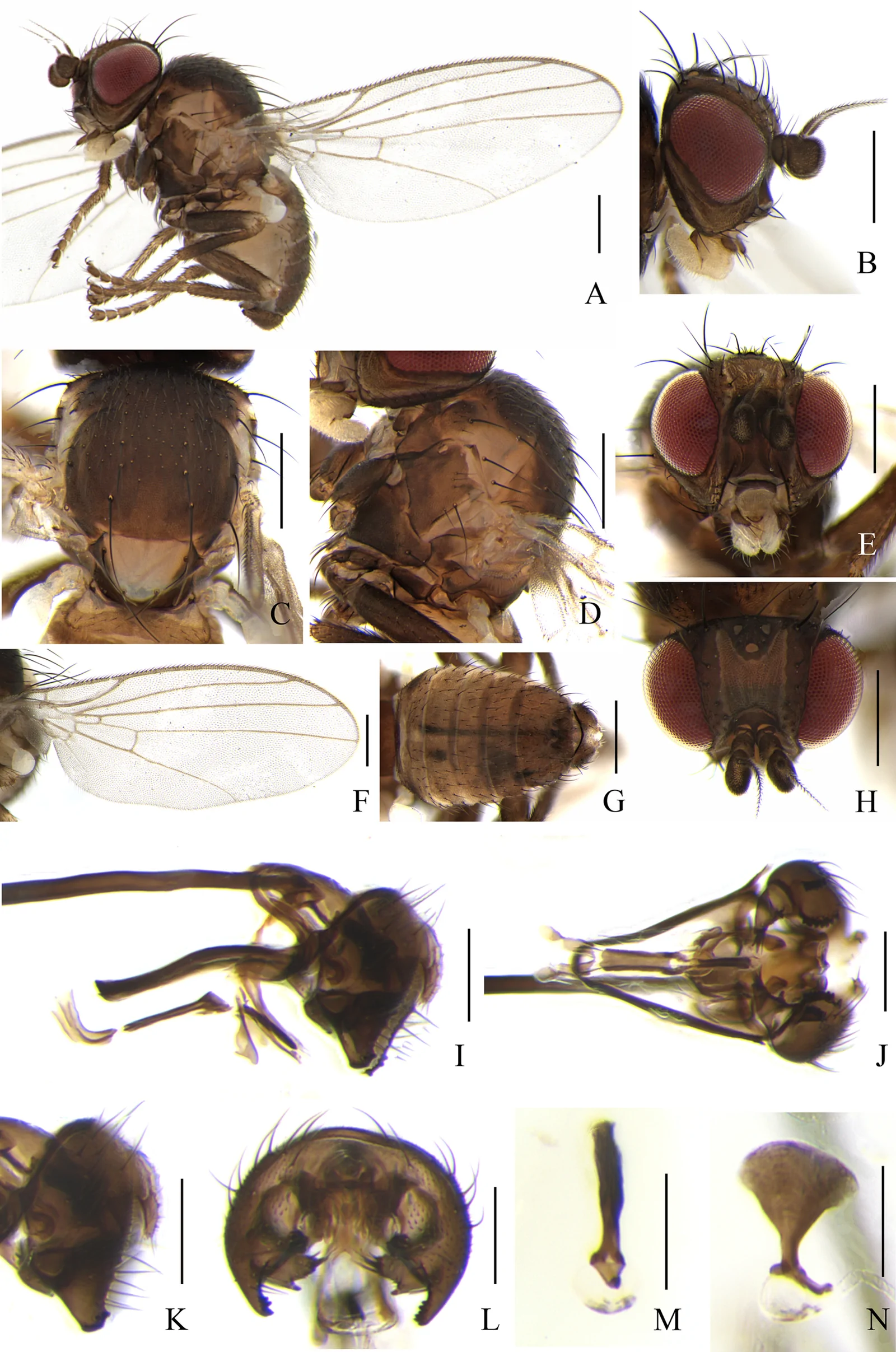
*Metopomyza laeta* Sasakawa, 1955. Male. **A**. Habitus, lateral view; **B, E, H**. Head, lateral, anterior, and dorsal view; **C, D**. Thorax, dorsal and lateral view; **F**. Wing; **G**. Abdomen, dorsal view; **I, J**. Genitalia, lateral and ventral view; **K, L**. Epandrial complex, lateral and posterior view; **M, N**. Ejaculatory apodeme, ventral and lateral view. Scale bars: 0.25 mm (**A–H**); 0.1 mm **(I–N**).

Wing length 1.6–1.8 mm. Head (Fig. 3B, E, H) brownish black; frons dirty yellow to brownish black. Gena deep, ~1/3 height of eye. Notopleuron to base of wing yellow (Fig. 3D). Legs black with all knees yellow. Wing (Fig. 3F) with ultimate and penultimate sections of M_4_ in proportion of 2.0–2.5:1. Calypter grayish yellow with margin and fringe deep black, sometimes dark brown. Abdomen dark brown on posterior margin and tergites 1–3 yellow on lateral margin. Male genitalia (Fig. 3I–N): epandrium (Fig. 3K, L) with 11–13 tubercle-like setae on ventral corner and 17–19 tubercle-like setae on inner surface (Fig. 3J); surstylus with five or six setae on anterodorsal corner and one or two tubercle-like setae on posteroventral corner; inner margin of paraphallus not expanded dorsally; distiphallus distinctly curved upward (Fig. 3I).

#### Chinese name.

Liang e qian ying (亮额潜蝇).

#### Distribution.

Palaearctic: China (Inner Mongolia)^#^, Czech Republic, France, Germany, Japan, Russia (East Siberia, Far East), Sweden.

#### Host.

Unknown.

#### Remarks.

The species is similar to *M. nigriorbita* in the following characters: fronto-orbital plate with 2 *ori* and 2 *ors*; thorax largely black, notopleuron yellow; anepisternum dark brown except for yellow dorsal and posterior margin; mesonotum with 0+3 *dc*, *acr* in 4 irregular rows. But it can be distinguished by the combination of characters: fronto-orbital plate dark brown, ~1/2 width of frons; mesonotum matt; calypter margin and fringe blackish or dark brown; inner margin of paraphallus relatively narrow, not expanded dorsally. In *M. nigriorbita*, fronto-orbital plate usually yellow, sometimes infuscate along inner margin of eye or largely black, ~1/3 width of frons; mesonotum moderately shiny; calypter margin and fringe whitish, sometimes paler; inner margin of paraphallus broadly expanded dorsally and almost reaching the base of mesophallus.

### Metopomyza scutellata

Taxon classificationAnimaliaDipteraAgromyzidae

(Fallén, 1823)

DB8F70A7-3FDD-528B-BF55-867DD5A6C1B1

**Figure 4.**
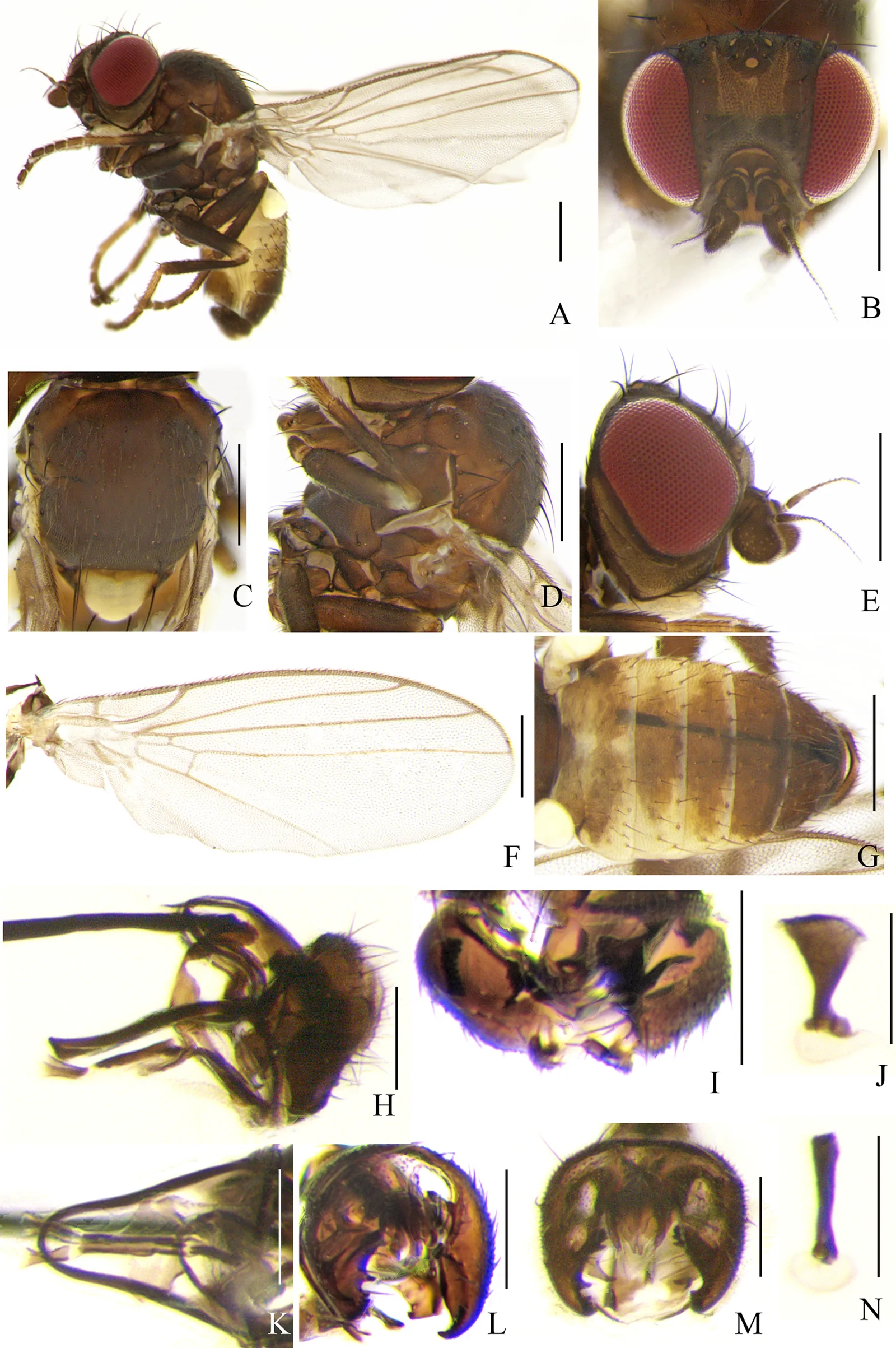
*Metopomyza scutellata* (Fallén, 1823). Male with abdomen mostly brown and tergites 2–4 yellowish laterally. **A**. Habitus, lateral view; **B, E**. Head, anterodorsal and lateral view; **C, D**. Thorax, dorsal and lateral view; **F**. Wing; **G**. Abdomen, dorsal view; **H**. Genitalia, lateral view; **I, L, M**. Epandrial complex, ventral, posterolateral and posterior view; **J, N**. Ejaculatory apodeme, lateral and ventral view; **K**. Phallic complex, ventral view. Scale bars: 0.25 mm (**A–G**); 0.1 mm (**H–N**).

**Figure 5.**
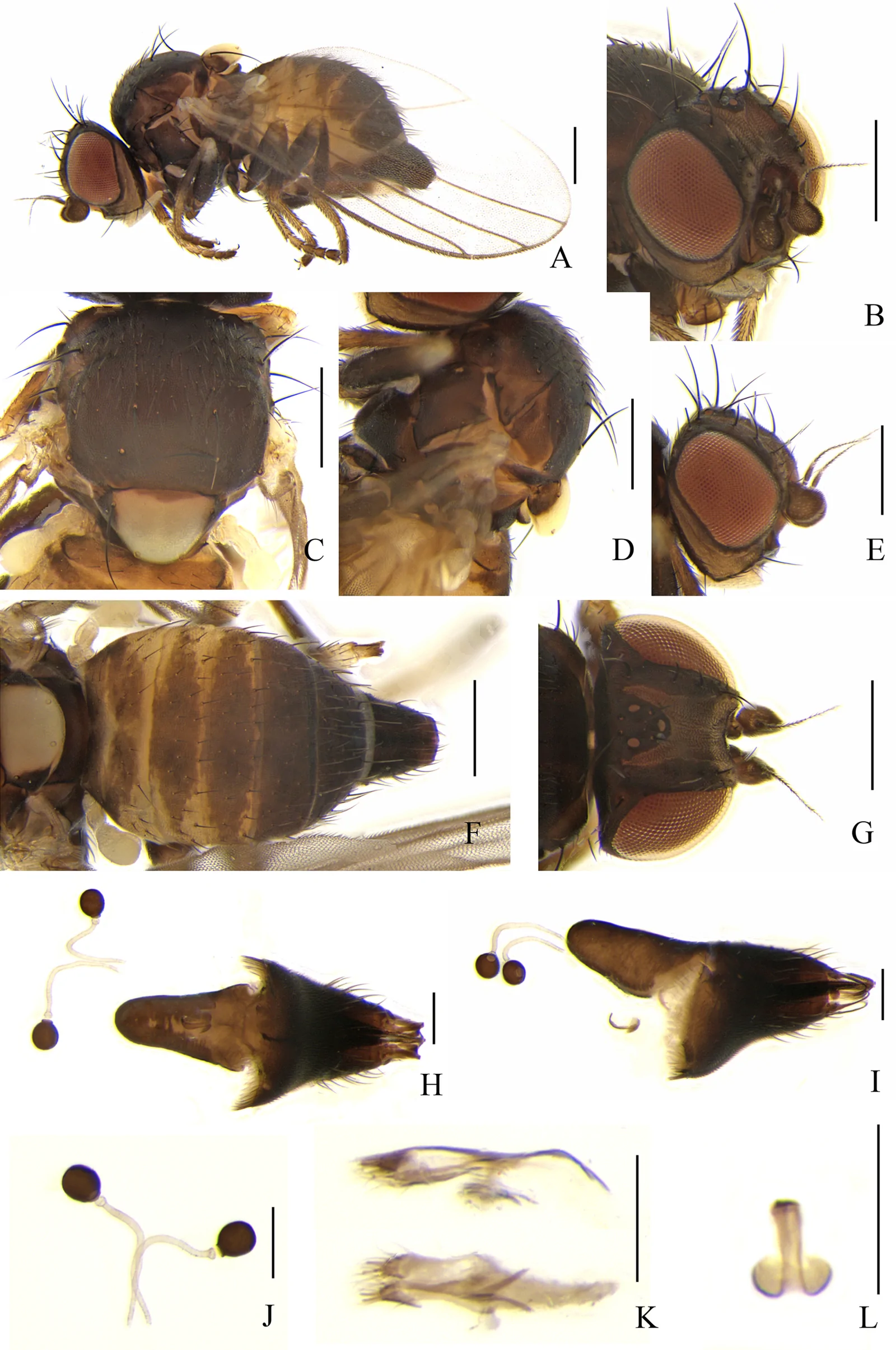
*Metopomyza scutellata* (Fallén, 1823). Female with abdomen mostly brown and tergites 2–4 yellowish laterally. **A**. Habitus, lateral view; **B, E, G**. Head, anterolateral, lateral, and dorsal view; **C, D**. Thorax, dorsal and lateral view; **F**. Abdomen, dorsal view; **H, I**. Spermathecae, ventral receptacle and oviscape, ventral and lateral view; **J**. Spermathecae; **K**. Proctiger, lateral and ventral view; **L**. Ventral receptacle, ventral view. Scale bars: 0.25 mm (**A–G**); 0.1 mm (**H–L**).

**Figure 6.**
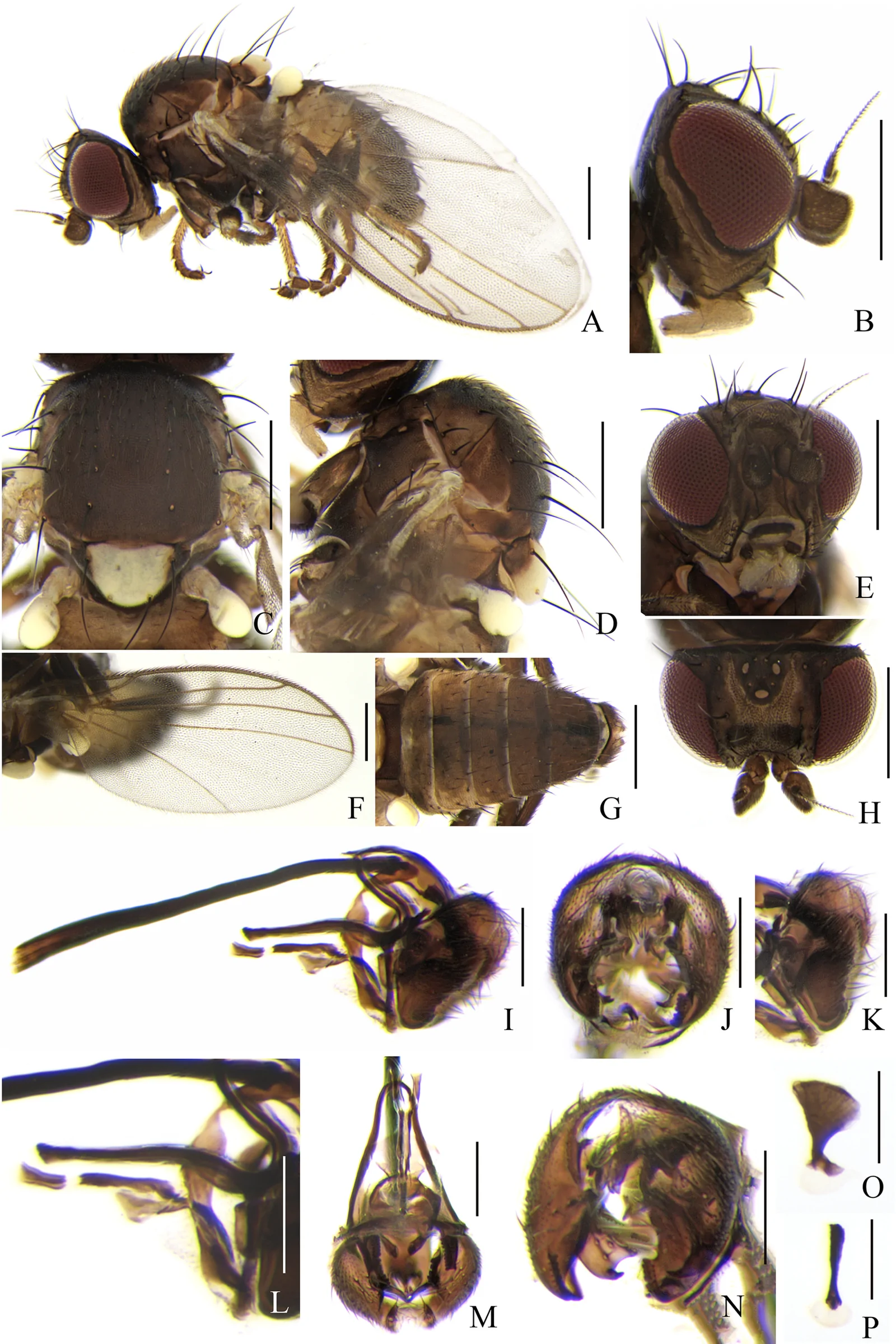
*Metopomyza scutellata* (Fallén, 1823). Male with abdomen mostly brown and tergites 1–4 yellow on posterior margin. **A**. Habitus, lateral view; **B, E, H**. Head, lateral, anterior, and dorsal view; **C, D**. Thorax, dorsal and lateral view; **F**. Wing; **G**. Abdomen, dorsal view; **I, M**. Genitalia, lateral and ventral view; **J, K, N**. Epandrial complex, posterior, lateral and posterolateral view; **L**. Phallic complex, lateral view; **O, P**. Ejaculatory apodeme, lateral and ventral view. Scale bars: 0.25 mm (**A–H**); 0.1 mm (**I–P**).

**Figure 7.**
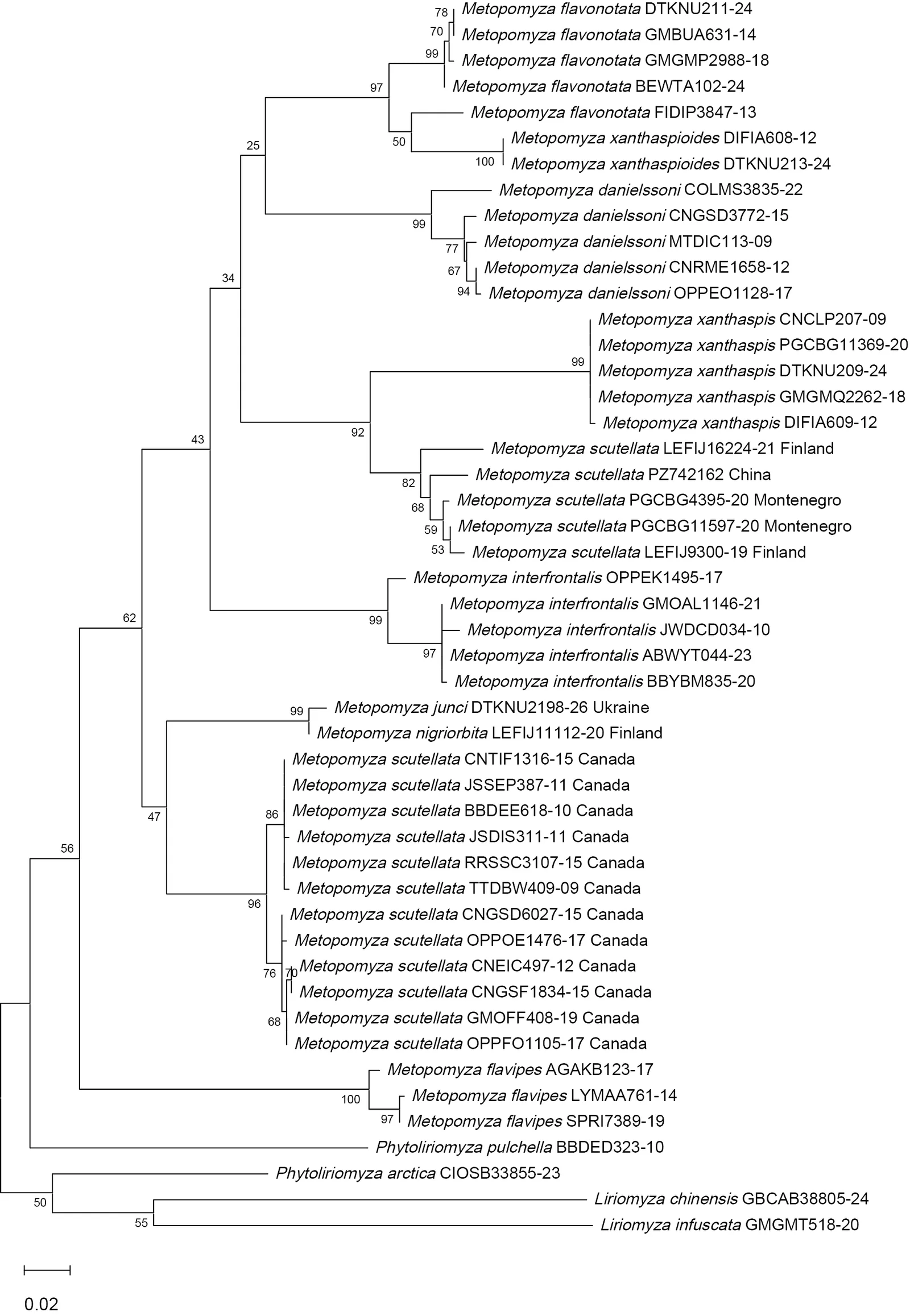
The Maximum-Likelihood (ML) phylogenetic tree of *Metopomyza* COI sequences.

**Figure 8.**
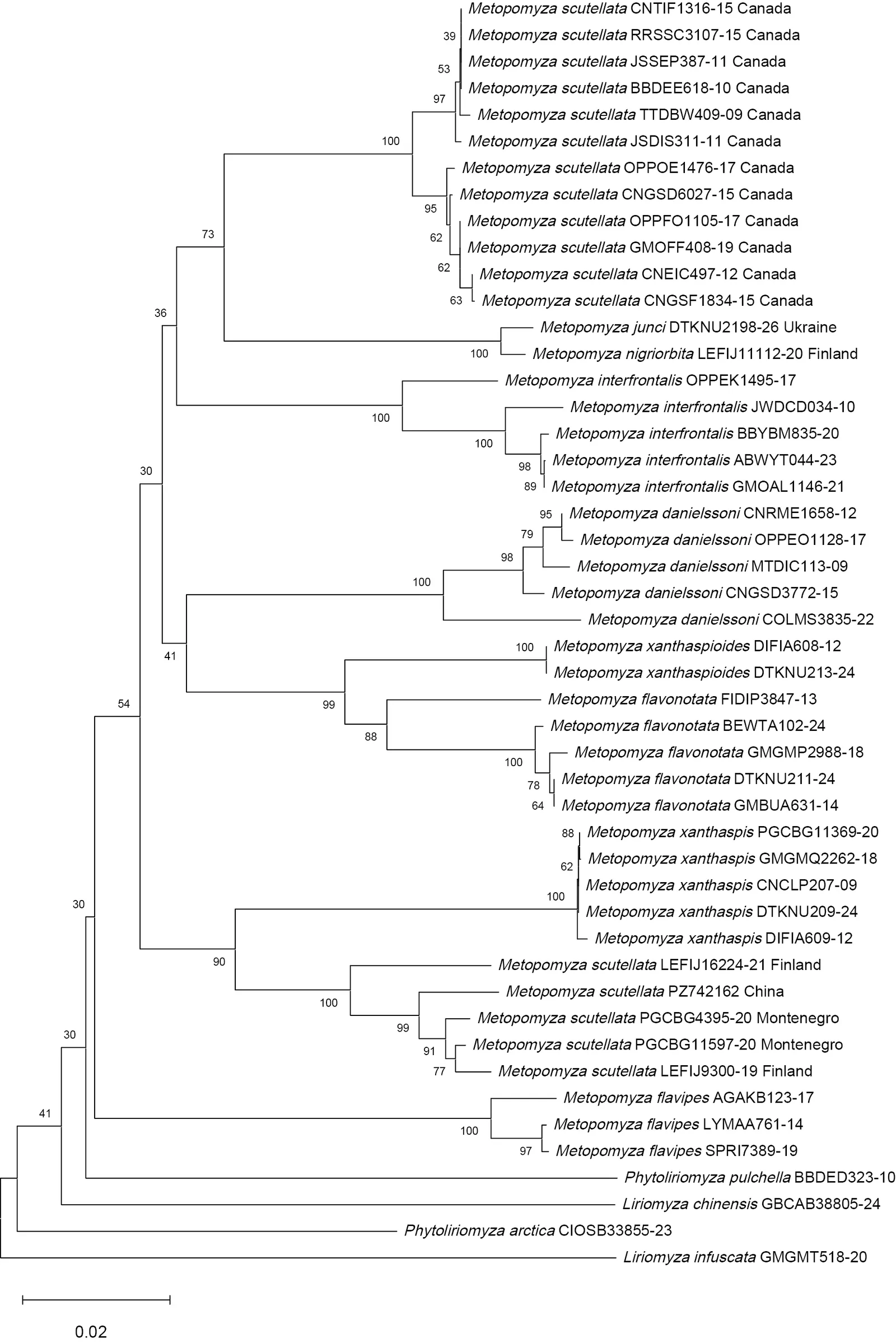
The Neighbor-Joining (NJ) phylogenetic tree of *Metopomyza* COI sequences.

Figs 4A–N, 5A–L, 6A–P, 7, 8

Agromyza scutellata Fallén, 1823: 7.
Liriomyza (Haplomyza) flavoscutellaris Zetterstedt, 1938 (*sensu*Hendel 1931: 222).
Metopomyza scutellata (Fallén, 1823): Spencer 1965: 253; 1976: 286; Zlobin 1995: 154; Černý 2018: 130; Černý et al. 2022: 424.

#### Specimens examined.

Specimens with abdomen mostly brown and tergites 1–4 narrowly yellow on posterior margin (Fig. 6G): • 2♂ (IMAU; 1♂ genitalia dissected), China, Inner Mongolia, Genhe City, Mangui Town, the northern primitive forest region of Greater Khingan Mountains, Aba River Third Branch Line, burned area in 2018, Pinus sylvestris var. mongholica malaise trap 36 (one meter above the ground), 52°21'07.00"N, 121°27'05.30"E, 646.3 m, 28.VII.2023, leg. Qin-Jianrong Liu.

Specimens with abdomen mostly brown and tergites 2–4 broadly yellow laterally (Figs 4G, 5F): China, Inner Mongolia, Genhe City, Mangui Town, the northern primitive forest region of Greater Khingan Mountains: • 1♂ (IMAU), Changliangbeishan houdu, burned area in 2002, *Pinus pumila*, malaise trap 3 (three meters above the ground), 52°25'31.24"N, 120°54'40.10"E, 1,009 m, 28.VII.2022, leg. Rui Ma, Qin-Jianrong Liu; • 1♂ (IMAU), Changliangbeishan houdu, burned area in 2002, Pinus sylvestris var. mongholica malaise trap 6 (one meter above the ground), 52°26'01.00"N, 120°56'53.20"E, 985 m, 14.VII.2022, leg. Li Shi, Zhi-wei Wang, Rui Ma; • 2♂1♀ (IMAU; 1♂ genitalia dissected), Changliangbeishan houdu, burned area in 2002, *Larix gmelinii*, malaise trap 2 (one meter above the ground), 52°25'31.24"N, 120°54'15.76"E, 1,032 m, 14.VII.2022, leg. Li Shi, Zhi-wei Wang, Rui Ma; • 2♀ (IMAU; 1♀ used for DNA extraction; DNA sequence number PZ742162 from GenBank), Aba River Third Branch Line, unburned area, Pinus sylvestris var. mongholica malaise trap 34 (one meter above the ground), 52°22'00.00"N, 120°27'07.00"E, 659 m, 28.VII.2022, leg. Rui Ma, Qin-Jianrong Liu; • 1♂ (IMAU; genitalia dissected), Aba River Third Branch Line, unburned area, Pinus sylvestris var. mongholica malaise trap 33 (three meters above the ground), 52°22'00.00"N, 121°27'07.00"E, 659 m, 28.VII.2022, leg. Rui Ma, Qin-Jianrong Liu; • 1♂ (IMAU), Wulonggan Forestry Center at 4207 meters, burned area in 2007, Pinus sylvestris var. mongholica malaise trap 17 (three meters above the ground), 52°51'53.10"N, 120°58'55.80"E, 823 m, 28.VII.2022, leg. Rui Ma, Qin-Jianrong Liu. • 2♀ (IMAU), China, Hebei Province, Chengde City, Weichang County, Saihanba National Forest Park, Dahuanqi Town, *Quercus mongolica*, malaise trap 6, 42°22'00.12"N, 117°36'59.45"E, 1,361 m, 09.VII.2023, leg. Qi Liu, Sheng Jin.

#### Diagnosis.

Wing length 1.4–1.5 mm. Head (Figs 4B, 4E, 5B, 5E, 5G, 6B, 6E, 6H) mostly brownish black; fronto-orbital plate slightly shiny, constricted anteriorly. Gena ~1/3 height of eye. Legs black with all knees contrastingly yellow. Wing (Figs 4F, 6F) with ultimate and penultimate sections of M_4_ in the ratio of 3:1 (*dm-m* sometimes lacking or incomplete). Abdomen mostly brown, with tergites 1–4 narrowly yellow on posterior margin (Fig. 6G), sometimes tergites 2–4 broadly yellow laterally (Figs 4G, 5F). Male genitalia (Figs 4H–N, 6I–P): epandrium (Figs 4I, 4L, 4M, 6J, 6K, 6N) with a row of stout tubercle-like setae along ventral margin, a tubercle-like seta and five or six small teeth-like processes on inner surface; surstylus with 4–6 setulae on inner surface and one or two tubercle-like setae at posterior corner (Figs 4L, 6N). Female terminalia (Fig. 5H–L): spermathecae dark brown, nearly circular; spermathecal duct weakly sclerotized, trumpet-shaped near spermathecae (Fig. 5J).

#### Chinese name.

Xiao dun e qian ying (小盾额潜蝇).

#### Distribution.

Palaearctic: Albania, Andorra, Belarus, Bulgaria, China (Inner Mongolia, Hebei)^#^, Croatia, Czech Republic, Denmark, Estonia, Finland, France, Germany, Georgia (Brice et al. 2026), Greece, Hungary, Iran, Italy, Kazakhstan, Kyrgyzstan, Latvia, Liechtenstein, Lithuania, Malta, Netherlands, Norway, Poland, Portugal, Russia (European part, East Siberia, Far East), Slovakia, Spain, Sweden, Switzerland, Tajikistan, United Kingdom, Turkey, Uzbekistan; Nearctic: Canada, USA.

#### Host.

*Carex* spp.

#### Remarks.

*Metopomyza scutellata* is morphologically almost indistinguishable from *M. junci*, with only the yellow lateral abdominal tergites of *M. junci* suggested as a diagnostic character by von Tschirnhaus (1981). However, Zlobin (1995) considered color characteristics of the abdomen unstable and unreliable for identification of the two species according to those specimens from the East Palaearctic, but the subsequent researchers still used the color characteristics of the abdomen for separating the two species (Papp and Černý 2017; Warrington and Perry 2020). Contrary to morphological classification, the host preferences of two species are clearly distinct. The host plants of *M. scutellata* are *Carex* spp. (Cyperaceae), which are widely distributed in temperate and wetland globally. The sampling habitat supports four *Carex* species: *Carex angarae* Steud., *C. meyeriana* Kunth, *C. pediformis* C. A. Mey. and *C. korshinskyi* Kom. The host plant of *M. junci* is *Juncus gerardii* Loisel. (Juncaceae) in von Tschirnhaus (1981), as the statement by Warrington and Perry (2020) reported “*M. junci* is known to utilize *Juncus*, chiefly *J. gerardii* Loisel., saltmarsh rush”. The sampling locality also harbors four *Juncus* species: *Juncus bufonius* L., *J. gracillimus* (Buchenau) V. I. Krecz. & Gontsch., *J. papillosus* Franch. & Sav., and *J. turczaninowii* (Buch.) V.I. Krecz. Unfortunately, our specimens were only collected with Malaise traps and not directly collected from the host plants.

The Maximum-Likelihood and Neighbor-Joining phylogenetic trees (Figs 7, 8) show that the species *M. scutellata* consists of two clades, one from the Palaearctic Region (China, Finland, Montenegro) and another from the Nearctic Region. Although *M. junci* is clustered with the Palaearctic clade of *M. scutellata*, it is first clustered with *M. nigriorbita* (Hendel, 1931) as a single clade.

The newly examined specimen of *M. scutellata* (accession number PZ742162) from Inner Mongolia, China—characterized by a predominantly brown abdomen and tergites 2–4 with broad lateral yellow markings—is nested within the Palaearctic clade of *M. scutellata*. ‌Moreover, our generated sequence shares up to 98% identity following alignment with publicly available *M. scutellata* sequences. ‌Based on the above analysis, it is confirmed that our specimens belong to *M. scutellata*, but this does not prove that *M. scutellata* and *M. junci* are conspecific.

##### Key to *Metopomyza* species worldwide (updated from Zlobin 2003)

**Table:** 

1	Notopleural triangle dark, black or brown	**2**
–	Notopleural triangle yellow	**14**
2	Femora entirely black or fore knees very narrowly and indistinctly yellowish brown	**3**
–	All femora black with yellow knees	**9**
3	Scutellum narrowly yellow medially, all margin adjoining mesonotum black	***M. knutsoni* Zlobin**
–	Scutellum largely yellow, black laterally to base of basal scutellar setae	**4**
4	Crossvein *dm-m* absent	**5**
–	Crossvein *dm-m* present	**6**
5	Wing length 1.3–1.5 mm; intra-alar setae absent; costa strongly extended to vein M_1_, veins R_4+5_ and M_1_ subequal in thickness	***M. xanthaspis* (Loew)**
–	Wing length 2.0–2.5 mm; intra-alar setae present; costa ending midway between veins R_4+5_ and M_1_ or M_1_ distinctly thinner than R_4+5_	***M. xanthaspioides* (Frey)**
6	Wing with the ultimate section of M_4_ distinctly more than twice as long as the penultimate one	**7**
–	Wing with the ultimate section of M_4_ at most twice as long as the penultimate one	**8**
7	Wing length 1.9–2.2 mm; mesonotum very shiny, with up to five differentiated postsutural *dc*	***M. flavonotata* (Haliday)**
–	Wing length 1.4–1.6 mm; mesonotum slightly matt or weakly shiny, with three postsutural *dc*	***M. danielssoni* Zlobin**
8	Frons with 1 *ors*; antenna entirely black	***M. nigripes* Spencer**
–	Frons with 2 *ors*; antenna with scape and pedicel black, first flagellomere yellow but testaceous at base	***M. nepalensis* Sasakawa**
9	Scutellum completely black	***M. nigrina* Zlobin**
–	Scutellum largely yellow	**10**
10	Wing with the ultimate section of M_4_ twice length of the penultimate one	***M. interfrontalis* (Melander)**
–	Wing with the ultimate section of M_4_ 3× length of the penultimate one	**11**
11	Mesonotum with a pair of presutural *dc*	***M. griffithsi* (Sehgal)**
–	Mesonotum without presutural *dc*	**12**
12	Abdomen entirely brownish black; male genitalia (Fig. 1I–N): hypophallus directed anteriorly; distiphallus conspicuously dilated on distal half, ~1/2 length of mesophallus	***M. hebeiensis* sp. nov**.
–	Abdomen brownish black with yellow lateral and posterior margins; male genitalia not as above	**13**
13	Host plant *Juncus*, chiefly *Juncus gerardii*, saltmarsh rush	***M. junci* Tschirnhaus**
–	Host plant *Carex* spp	***M. scutellata* (Fallén)**
14	Antenna with first flagellomere bright yellow	**15**
–	Antenna with first flagellomere at least partly brown	**17**
15	Wing with crossvein *dm-m* absent	***M. henshawi* Zlobin**
–	Wing with crossvein *dm-m* present	**16**
16	Legs black but all knees bright yellow; abdomen largely black but front tergites laterally yellow	***M. nigrohumeralis* (Hendel)**
–	Legs dirty yellow, fore tibiae and all tarsi pale brown; abdomen largely yellow but tergite 1 pale brown, tergites 2–5 each with pale brown band on basal 1/4–1/3	***M. lingulata* Sasakawa & Çikman**
17	Wing with crossvein *dm-m* absent or incomplete	***M. adretana* Boucher & Wheeler**
–	Wing with crossvein *dm-m* present and complete	**18**
18	Fronto-orbital plates strongly projecting above eye; gena forming an unusually broad ring below eye	**19**
–	Fronto-orbital plates narrowly projecting above eye; gena forming a narrow ring below eye	**20**
19	Frons with 1 *ors* and 4–5 *ori*; mesonotum with 1+4 *dc*	***M. antiqua* Zlobin**
–	Frons with 2 *ors* and 3 *ori*; mesonotum with 1+3 *dc*	***M. flavipes* Spencer**
20	Wing with the ultimate section of M_4_ slightly longer than the penultimate one; mesonotum with *acr* in two sparse rows	***M. curtifistula* Sasakawa**
–	Wing with the ultimate section of M_4_ 2.0–2.5 times as long as the penultimate one; mesonotum with *acr* at least in four rows	**21**
21	Gena narrow, 1/8–1/7 height of eye; vibrissal angle broadly rounded; male genitalia: mesophallus very short, ~1/2 length of distiphallus; distiphallus nearly straight (Zlobin 1995: fig. 63)	***M. levis* Zlobin**
–	Gena deep, 1/5–1/3 height of eye; vibrissal angle more or less angulate; male genitalia: mesophallus long, 1.2–1.5× length of distiphallus; distiphallus distinctly curved (Fig. 3I)	**22**
22	Fronto-orbital plate usually yellow, sometimes infuscate along inner margin of eye or largely black (Zlobin 1995: fig. 46); frontal vitta bright yellow; calypter margin and fringe whitish, sometimes paler, brownish yellow; male genitalia: inner margin of paraphallus broadly extended dorsally (Zlobin 1995: fig. 47)	***M. nigriorbita* (Hendel)**
–	Fronto-orbital plate usually deep black, slightly shiny (Fig. 3H); frontal vitta dirty yellow to brownish black; calypter margin and fringe deep black, sometimes dark brown; male genitalia: inner margin of paraphallus not extended dorsally (Fig. 3I; Zlobin 1995: fig. 56)	***M. laeta* Sasakawa**

##### Checklist of twenty-three worldwide *Metopomyza* species

**1. *Metopomyza adretana* Boucher & Wheeler, 2001**

**Literature**. Boucher and Wheeler 2001.

**Distribution**. Nearctic: Canada.

**2. *Metopomyza antiqua* Zlobin, 1983**

**Literature**. Zlobin 1983, 1995.

**Distribution**. Palaearctic: Russia (East Siberia).

**3. *Metopomyza curtifistula* (Sasakawa, 1996) (as *Phytoliriomyza* )**

**Literature**. Sasakawa 1996; Zlobin 2007a.

**Distribution**. Oriental: Nepal.

**4. *Metopomyza danielssoni* Zlobin, 1995**

**Literature**. Zlobin 1995.

**Distribution**. Nearctic: Canada (https://www.gbif.org/), USA.

**5. *Metopomyza flavipes* Spencer, 1969**

**Literature**. Spencer 1969.

**Distribution**. Nearctic: Canada.

**6. *Metopomyza flavonotata* (Haliday, 1833) (as *Agromyza* )**

**Literature**. Sasakawa 1955; Spencer 1976; Soós and Papp 1984; Černý 2005; Andersen 2012; Clemons 2015; Černý 2021; Černý et al. 2022.

**Distribution**. Palaearctic: Austria, Belarus, Belgium (Scheirs et al. 1995), Bulgaria (Černý et al. 2022), Czech Republic (Černý 2021), Denmark, Estonia, Finland, France, Germany, Hungary, Ireland, Italy, Japan (Sasakawa 1955), Latvia, Lithuania, Netherlands, Norway (Andersen 2012), Romania, Russia (European part, Siberia, Far East), Slovakia, Sweden, Switzerland (Černý 2005), United Kingdom (Clemons 2015).

**7. *Metopomyza griffithsi* (Sehgal, 1971)**

**Literature**. Sehgal 1971; Spencer 1981; Zlobin 1995.

**Distribution**. Nearctic: Canada, USA.

**8. *Metopomyza hebeiensis* sp. nov**.

**Distribution**. Palaearctic: China (Hebei).

**9. *Metopomyza henshawi* Zlobin, 2003**

**Literature**. Zlobin 2003.

**Distribution**. Palaearctic: Russia (East Siberia).

**10. *Metopomyza interfrontalis* (Melander, 1913) (as *Agromyza* )**

*Liriomyza xanthaspida* Hendel, 1920 (synonym).

**Literature**. Spencer 1981; Černý 2009a; Kahanpää 2014.

**Distribution**. Palaearctic: Austria (Franz 1989), Czech Republic (Černý 2009a), Denmark, Estonia, Finland (Kahanpää 2014), Latvia, Lithuania, Norway, Poland, Russia (European part, Polar Urals, Siberia, Far East), Slovakia, Sweden (https://www.gbif.org/); Nearctic: Canada, USA.

**11. *Metopomyza junci* von Tschirnhaus, 1981**

**Literature**. Papp and Černý 2017; Černý 2018; Warrington and Perry 2020; Černý et al. 2020.

**Distribution**. Palaearctic: Austria, Denmark, Germany, Hungary (Papp and Černý 2017), Iran (Černý et al. 2020), Kyrgyzstan, Lithuania, Netherlands, Poland, Portugal (Gil-Ortiz et al. 2011), Russia (Siberia), Slovakia, United Kingdom.

**12. *Metopomyza knutsoni* Zlobin, 1995**

**Literature**. Zlobin 1995.

**Distribution**. Palaearctic: Russia (European part).

**13. *Metopomyza laeta* Sasakawa, 1955**

**Literature**. Sasakawa 1955; Černý 2018.

**Distribution**. Palaearctic: China (Inner Mongolia)^#^, Czech Republic, France, Germany, Japan, Russia (East Siberia, Far East), Sweden (https://www.gbif.org/).

**14. *Metopomyza levis* Zlobin, 1995**

**Literature**. Zlobin 1995.

**Distribution**. Palaearctic: Russia (European part).

**15. *Metopomyza lingulata* Sasakawa & Çikman, 2011**

**Literature**. Çikman and Sasakawa 2011.

**Distribution**. Palaearctic: Turkey.

**16. *Metopomyza nepalensis* Sasakawa, 1996**

**Literature**. Sasakawa 1996.

**Distribution**. Oriental: Nepal.

**17. *Metopomyza nigrina* Zlobin, 1995**

**Literature**. Zlobin 1995.

**Distribution**. Palaearctic: Russia (Far East).

**18. *Metopomyza nigriorbita* (Hendel, 1931) (as *Liriomyza* )**

**Literature**. Spencer 1976; Soós and Papp 1984; Zlobin 1995; Černý 2005; Černý et al. 2022.

**Distribution**. Palaearctic: Austria, Belarus, Bulgaria (Černý et al. 2022), Czech Republic, Denmark, Estonia, Finland, France, Hungary, Italy, Japan, Latvia, Lithuania, Poland, Russia (European part, Far East), Slovakia, Sweden, Switzerland (Černý 2005), United Kingdom, Ukraine (https://www.gbif.org/), Uzbekistan.

**19. *Metopomyza nigripes* Spencer, 1981**

**Literature**. Spencer 1981.

**Distribution**. Nearctic: USA.

**20. *Metopomyza nigrohumeralis* (Hendel, 1931) (as *Liriomyza* )**

**Literature**. Spencer 1976; Soós and Papp 1984; Papp and Černý 2017; Telfer and Gibbs 2017.

**Distribution**. Palaearctic: Austria, Czech Republic, Denmark, Germany, Hungary, Italy, Russia (East Siberia), Slovakia, Sweden, United Kingdom.

**21. *Metopomyza scutellata* (Fallén, 1823) (as *Agromyza* )**

*Metopomyza flavoscutellaris* Zetterstedt, 1838 (synonym).

**Literature**. Spencer 1976; Soós and Papp 1984; Zlobin 1995; Černý 2005, 2007, 2009b, 2011, 2018; Černý and Merz 2006; Karpa 2007; Andersen 2012; Černý et al. 2018, 2022.

**Distribution**. Palaearctic: Albania, Andorra (Černý 2007), Belarus, Bulgaria, China (Inner Mongolia, Hebei)^#^, Croatia (Černý 2009b), Czech Republic, Denmark, Estonia, Finland, France, Georgia (Brice et al. 2026), Germany, Greece (Černý 2011), Hungary, Iran (Černý 2018), Italy, Kazakhstan, Kyrgyzstan, Latvia (Karpa 2007), Liechtenstein, Lithuania, Malta, Netherlands, Norway (Andersen 2012), Poland, Portugal (Černý et al. 2018), Russia (European part, East Siberia, Far East), Slovakia, Spain, Sweden, Switzerland (Černý 2005), Tajikistan, United Kingdom, Turkey, Uzbekistan; Nearctic: Canada, USA (https://www.gbif.org/).

**22. *Metopomyza xanthaspioides* (Frey, 1946) (as Liriomyza (Haplomyza))**

*Metopomyza anomala* Rohdendorf-Holmanova, 1960 (synonym).

**Literature**. Soós and Papp 1984; Andersen 2012; Černý 2021.

**Distribution**. Palaearctic: Czech Republic (Černý 2021), Estonia, Finland, France, Germany, Greece, Hungary, Lithuania, Norway (Andersen 2012), Russia (European part, East Siberia), Sweden, Switzerland, Ukraine (https://www.gbif.org/).

**23. *Metopomyza xanthaspis* (Loew, 1858) (as *Phytomyza* )**

**Literature**. Soós and Papp 1984; Scheirs et al. 1995; Spencer 1972; Černý 2005; Andersen 2018; Černý et al. 2020.

**Distribution**. Palaearctic: Albania (Černý et al. 2020), Andorra, Austria, Belgium (Scheirs et al. 1995), Bulgaria, China (Xinjiang), Croatia, Czech Republic, Denmark, Estonia, Finland, France, Germany, Hungary, Italy, Japan (Černý 2005), Kazakhstan, Latvia, Lithuania, Montenegro, Netherlands, Norway (Andersen 2018), Poland, Russia (European part, East Siberia, Far East), Slovakia, Spain, Sweden, Switzerland (Černý 2005), United Kingdom (https://www.gbif.org/), Turkey.

## Supplementary Material

XML Treatment for Metopomyza hebeiensis

XML Treatment for Metopomyza laeta

XML Treatment for Metopomyza scutellata

## Additional information

### Artificial Intelligence (AI) use

The authors accept full responsibility for the content of the manuscript, including the disclosure of any use of AI.

No AI tools were used in the preparation of this manuscript.

### Author ORCIDs

X.-T. Fu https://orcid.org/0009-0002-3970-8367

L. Shi https://orcid.org/0000-0001-5842-4904

### Data availability

All of the data that support the findings of this study are available in the main text or Supplementary Information.

## Supplementary materials

Supplementary material 1 List of 48 sequences Data type fasta https://binary.pensoft.net/file/1747697This dataset is made available under the Open Database License (http://opendatacommons.org/licenses/odbl/1.0/). The Open Database License (ODbL) is a license agreement intended to allow users to freely share, modify, and use this Dataset while maintaining this same freedom for others, provided that the original source and author(s) are credited. Xin-Ting Fu, Li Shi
